# Comparison of two different starting dose of rhFSH in GnRH antagonist protocol for patients with normal ovarian reserve

**DOI:** 10.3389/fendo.2023.1068141

**Published:** 2023-01-20

**Authors:** Zhi-cheng Jia, Yong-qian Li, Ran Li, Sen Hou, Qing-chang Xia, Kai Yang, Pei-xuan Wang, Shu-miao Li, Zhen-gao Sun, Ying Guo

**Affiliations:** ^1^ The First Clinical College, Shandong University of Traditional Chinese Medicine, Jinan, China; ^2^ College of Traditional Chinese Medicine, Shandong University of Traditional Chinese Medicine, Jinan, China; ^3^ Reproductive and Genetic Center of Integrative Medicine, Affiliated Hospital of Shandong University of Traditional Chinese Medicine, Jinan, China; ^4^ The Second Clinical College, Beijing University of Chinese Medicine, Beijing, China

**Keywords:** GnRH antagonist protocol, starting dose of rhFSH, live birth rate, normal ovarian reserve, *in vitro* fertilization

## Abstract

**Objective:**

To evaluate different starting doses of recombinant human follicle-stimulating hormone (rhFSH) on pregnancy outcomes for patients with normal ovarian reserve during gonadotropin- releasing hormone antagonist (GnRH-ant) protocol-controlled ovarian stimulation of *in vitro* fertilization (IVF) cycles.

**Methods:**

In this retrospective study, a total of 1138 patients undergoing IVF cycles following the GnRH-ant protocol were enrolled. Patients were divided into two groups according to the starting dose of rhFSH. 617 patients received a starting dose of rhFSH of 150 IU, and 521 patients received a starting dose of rhFSH of 225 IU. We compared demographic characteristics, ovarian stimulation and embryological characteristics, and pregnancy and birth outcomes between the two groups. Multivariate logistic regression analysis was performed to examine the possible effects of the known potential confounding factors on pregnancy outcomes.

**Results:**

The number of oocytes retrieved in the 150 IU rhFSH group was significantly lower than those in the 225 IU rhFSH group. There was no significant difference between the two groups referring to embryological characteristics. The proportion of fresh embryo transfer in the 150 IU rhFSH group was significantly higher than that in the 225 IU rhFSH group (48.30% vs. 40.90%), and there was no difference in the risk of ovarian hyperstimulation syndrome and pregnancy outcomes between the two groups.

**Conclusions:**

In conclusion, the starting dose of rhFSH of 150 IU for ovarian stimulation has a similar pregnancy outcome as starting dose of rhFSH of 225 IU in GnRH-ant protocol for patients with normal ovarian reserve. Considering the potential cost-effectiveness and shorter time to live birth, the starting dose of rhFSH of 150 IU may be more suitable than 225 IU.

## 1 Introduction

Gonadotropin-releasing hormone agonist (GnRH-a) protocol and gonadotropin-releasing hormone antagonist (GnRH-ant) protocol have been widely applied in controlled ovarian stimulation (COS) for *in vitro* fertilization (IVF)/intracytoplasmic sperm injection (ICSI) cycles; they are comparable in clinical outcomes, obstetric and perinatal outcomes (1). GnRH-ant protocol, which was discovered in the 1990s, is increasingly favored in clinical practice because of its physiological advantages (2). Compared with the GnRH-a protocol, the GnRH-ant protocol competitively binds to the receptor of the pituitary gland and causes rapid suppression of gonadotropin release without “flare-up” effect. Meanwhile, the GnRH-ant protocol can effectively reduce the consumption of gonadotropin and greatly shorten the treatment time, and reduce the risk of ovarian hyperstimulation syndrome (OHSS) (3). Recombinant human follicle-stimulating hormone (rhFSH) is the key hormone that stimulates the development of multiple follicles during COS to obtain an adequate number of oocytes and embryos (4). Individualization of the starting dose of rhFSH is considered standard clinical practice. The optimum dose of rhFSH required to exceed the “FSH threshold” during the therapeutic “FSH window”, which is an inconclusive medication management, ranges from 100 IU to 300 IU (5). Starting dose of rhFSH selection mainly depends on the patient’s characteristics and ovarian reserve, which is affected by various biomarkers, including basal follicle-stimulating hormone (FSH), antral follicle count (AFC), and anti-Müllerian hormone (AMH) (6, 7). Therefore, a starting dose of rhFSH selection is often dependent on the clinical experience of specialists.

Classification of patients according to their ovarian reserve is the basis for selecting an appropriate starting dose of rhFSH. In 2022, the Chinese Medical Doctor Association (CMDA) promulgated the “Expert Consensus on Standardized Application of gonadotropin-releasing hormone antagonist in Assisted Reproductive Technology” (8), which divides the population into normal ovarian reserve (NOR), high ovarian reserve (HOR), diminished ovarian reserve (DOR), according to ovarian reserve. Patients meeting the following criteria were defined as NOR in the consensus, including age < 35 years; basal FSH level < 10 IU/L; AMH level 1.1-4.0 ng/L; and AFC 7-15. For NOR patients, the main treatment goals are to reduce the time to ovulation induction, shorten the time to live birth and increase the pregnancy rate of fresh embryo transfer. The recommended starting dose of rhFSH in the consensus is 150-225 IU for the NOR patients. Generally, the number of oocytes retrieved depends on the dose of rhFSH (9). However, individual women’s responses vary (10). GnRH-ant protocol was only used in China in 2013, and clinical experience is relatively lacking. The starting dose of rhFSH of 150 or 225 IU is a broad dose range, and it is unclear whether there is a difference in pregnancy outcomes between the two starting doses of rhFSH. This study aimed to investigate whether IVF and pregnancy outcomes would change in the NOR patients between the two starting doses of rhFSH.

## 2 Material and methods

### 2.1 Patients

Individuals who completed an autologous IVF/ICSI cycle and received a GnRH-ant protocol treated at the authors’ reproductive clinic from January 2014 to June 2021 were included in this single-center retrospective cohort study. The study was authorized by the local institutional review board (Reproductive Ethics Committee of The Affiliated Hospital of Shandong University of Traditional Chinese Medicine, approval no. SDTCM/E2204-02, dated April 2, 2022) and was undertaken at a public tertiary referral university hospital.

The inclusion criteria were as follows: (a) age < 35 years; (b) FSH level < 10 IU/L; (c) AMH level 1.1-4.0 ng/L; (d) basal AFC 7-15.

The exclusion criteria were as follows: (a) patients with polycystic ovary syndrome; (b) patients with ovarian insufficiency; (c) body mass index (BMI) > 28; (d) patients with an abnormal uterine cavity that affected embryo implantation; (e) patients with recurrent implantation failure; (f) patients who required a genetic diagnosis before embryo implantation.

**Figure 1:**
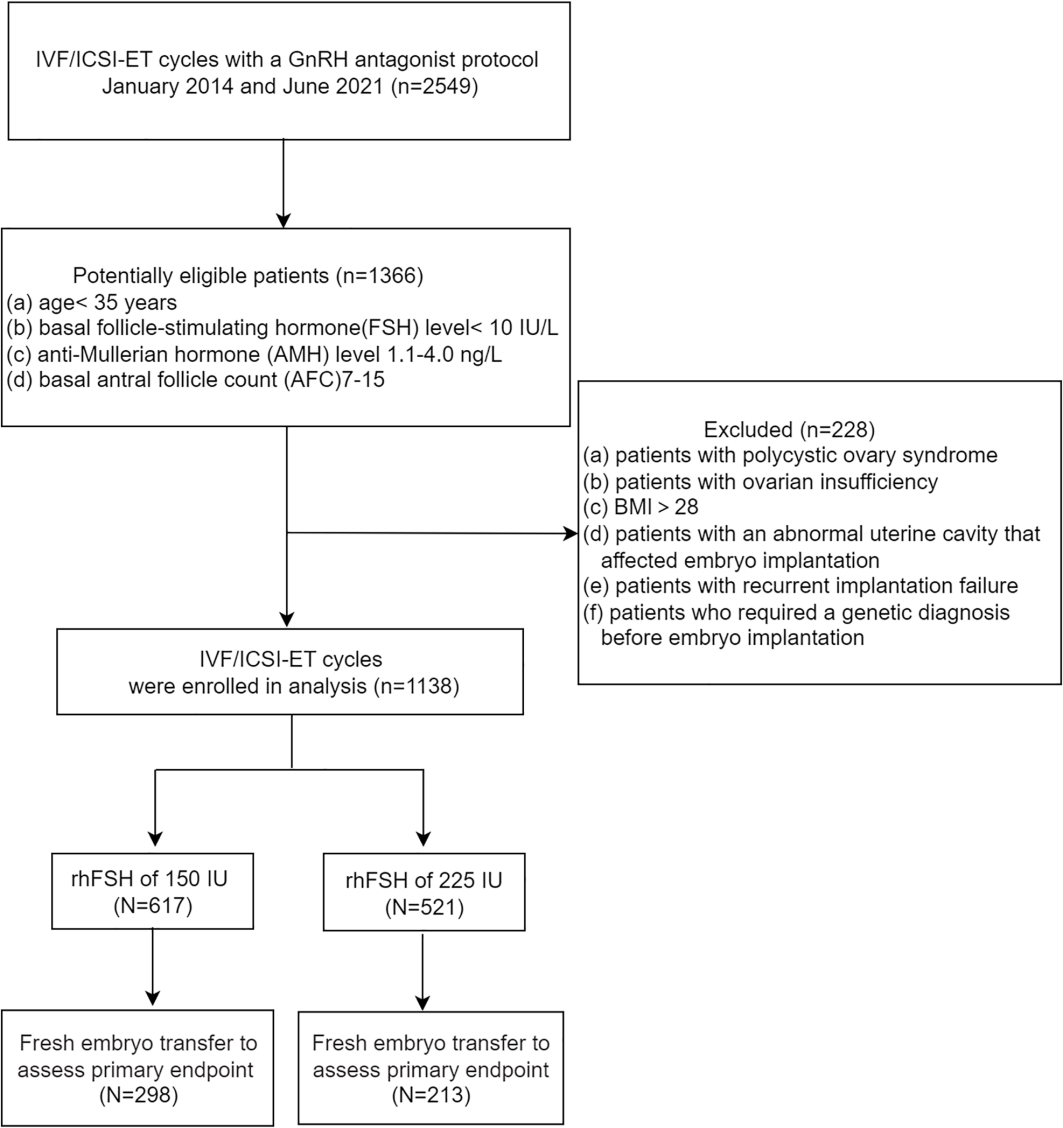
Flowchart of this study.

A total of 1138 patients were included in this study. Patients were divided into two groups according to the starting dose of rhFSH. 617 patients received a starting dose of 150 IU rhFSH, and 521 patients received a starting dose of 225 IU rhFSH (**Figure 1**).

### 2.2 Controlled ovarian stimulation protocols

All participants underwent IVF/ICSI treatment using a GnRH-ant protocol. Recombinant human follicle stimulating hormone (Gonal-F, Merck Serono, Switzerland) is administered on the second or third day of the menstrual cycle at a dose of 100-300 IU per day, depending on the woman’s age, FSH, AFC, and AMH.

During COS, participants were monitored for follicular recruitment and growth and endometrial thickness by serial transvaginal ultrasound and blood hormone tests, including estradiol (E2), progesterone (P4) and luteinizing hormone (LH) plasma levels. The dose of rhFSH may be increased or decreased according to the follicular development of the patient during COS, within the range of 50 IU. Cetrorelix (Merck Serono, Switzerland) of 0.25 mg/day was initiated until the trigger day, when the dominant follicle diameter was ≥14 mm, E2 ≥400 pg/ml. Human chorionic gonadotropin (hCG, Lizhu, Zhuhai, China) or GnRH agonist (triptorelin acetate; France) combined hCG (dual trigger) was administered to trigger the maturation of oocytes when there were three follicles measuring 18 mm or more in diameter.

Oocyte pick-up (OPU) was performed by transvaginal ultrasound-guided needle aspiration 35-36 hours following triggering, followed by standard IVF/ICSI as previously reported (11). Fresh embryo transfer was carried out 3 days (cleavage embryo) or 5 days (blastocyst) after OPU. Whole embryos were frozen if patients with a high risk of OHSS, high progesterone level (progesterone ≥1.5-2 ng/ml), severe hydrosalpinx, or endometrial polyp. Embryo grading was done following the proceedings of the Istanbul consensus (12). High-quality embryos were characterized as those that reached at least the six-cell stage with<20% fragmentation. Oral progesterone combined vaginal progesterone or intramuscular progesterone was used for luteal support since the day of OPU for fresh transfer cycles.

### 2.3 Outcome measures

We compared IVF and pregnancy outcomes at a different starting dose of rhFSH. Our primary outcome measure was live birth, which we defined as the delivery of at least one infant with breathing and heartbeat, regardless of gestational age. The secondary outcomes included positive pregnancy rate, biochemical pregnancy rate, clinical pregnancy rate, ectopic pregnancy rate, ongoing pregnancy rate, miscarriage rate, live birth rate and IVF outcomes. Positive pregnancy is defined as a serum-hCG level of at least 10 mIU/mL. Biochemical pregnancy loss is described as undetected pregnancy losses that are recorded only *via* a positive pregnancy test (serum hCG level 10 mIU/mL). After 10 gestational weeks, clinical pregnancy is defined as an intrauterine gestational sac with fetal heartbeat identified through transvaginal ultrasonography. Ectopic pregnancy is described as a pregnancy that occurs outside the uterine cavity. We thereby define ongoing pregnancy as a viable intrauterine pregnancy of at least 12 weeks, confirmed on an ultrasound scan (13). Miscarriage refers to the termination of pregnancy before 28 weeks of gestation and a fetus weighing less than 1000 g (14). OHSS was diagnosed according to the latest classification criteria (15).

### 2.4 Statistical analysis

All statistical analyses were performed with the SPSS 25.0 statistical software (IBM, Chicago, IL, USA). The K-S test was used for the normality test. Continuous variables are expressed as mean ± SD or median (IQR), and Categorical variables are expressed as number (n) and percentage (%). Mann-Whitney U test or Student’s t-tests were used for continuous variables, and the Chi-square test was used for categorical variables. Various factors affecting clinical outcomes were identified by univariate logistic regression analysis. Multivariate logistic regression analysis was performed to examine the possible effects of the following known potential confounding factors on pregnancy outcomes, including age, BMI, AMH, infertility type (primary or secondary), starting dose of rhFSH, ovulation trigger protocol, the total dose of Cetrorelix. A P-value < 0.05 was considered statistically significant.

## 3 Result

### 3.1 Baseline characteristics of the study population

A total of 1138 ovarian stimulation cycles with GnRH-ant protocol were included in this study, including 617 cycles a starting dose of rhFSH of 150 IU and 521 cycles with 225 IU. Patients’ baseline characteristics are detailed in **Table 1**. There were no statistically significant differences in the Mean age, duration of infertility, basal FSH, LH, E2, P4, AMH level, gravidity, parity, miscarriage, BMI, AFC, etiology of infertility, types of infertility between the two groups (all P > 0.05). The method of fertilization was similar between the two groups (P > 0.05).

**Table 1:** Comparison of general data between the two groups.

Groups	150 IU (n = 617)	225 IU (n = 521)	P-value
Age (years)	30.66 ± 3.07	30.75 ± 3.13	0.63
Infertility duration(years)	3.27 ± 2.1	3.23 ± 2.13	0.75
Basal FSH level (IU/L)	6.85 ± 1.48	6.69 ± 1.45	0.06
Basal LH level (IU/L)	5.34 ± 2.69	5.25 ± 2.57	0.59
Basal E2 level (pg/ml)	35.28 ± 9.44	35.01 ± 9.18	0.63
Basal P4 level (ng/ml)	0.91 ± 1.14	0.95 ± 1.31	0.61
AMH (ng/ml)	2.58 ± 0.85	2.61 ± 0.79	0.59
Gravidity (n)	0 (0,1)	0 (0,1)	0.89
Parity (n)	0 (0,0)	0 (0,0)	0.76
Miscarriage (n)	0 (0,0)	0 (0,0)	0.89
BMI (kg/m^2^)	23.32 ± 2.63	23.6 ± 2.54	0.07
Antral follicle count	11.18 ± 2.56	11.09 ± 2.51	0.53
Etiology of infertility			0.552
Tubal factor (%)	456/617 (73.90%)	370/521 (71.00%)	
Male factor (%)	121/617 (19.60%)	114/521 (21.90%)	
Others (%)	40/617 (6.50%)	37/521 (7.10%)	
Types of infertility			0.503
primary infertility	311/617 (50.40%)	273/521 (52.40%)	
secondary infertility	306/617 (49.60%)	248/521 (47.60%)	
Method of fertilization			0.346
ICSI	121/617 (19.60%)	114/521 (21.90%)	
IVF	496/617 (80.40%)	407/521 (78.10%)	

FSH, follicle stimulating hormone; LH, luteinizing hormone; E2, estradiol; P4, progesterone; AMH, anti-Müllerian hormone; BMI, body mass index; ICSI, intracytoplasmic single sperm injection; IVF, in vitro fertilization; Data are presented as mean ± SD, median (IQR) and n (%).

### 3.2 Ovarian stimulation outcomes

**Figure 2:**
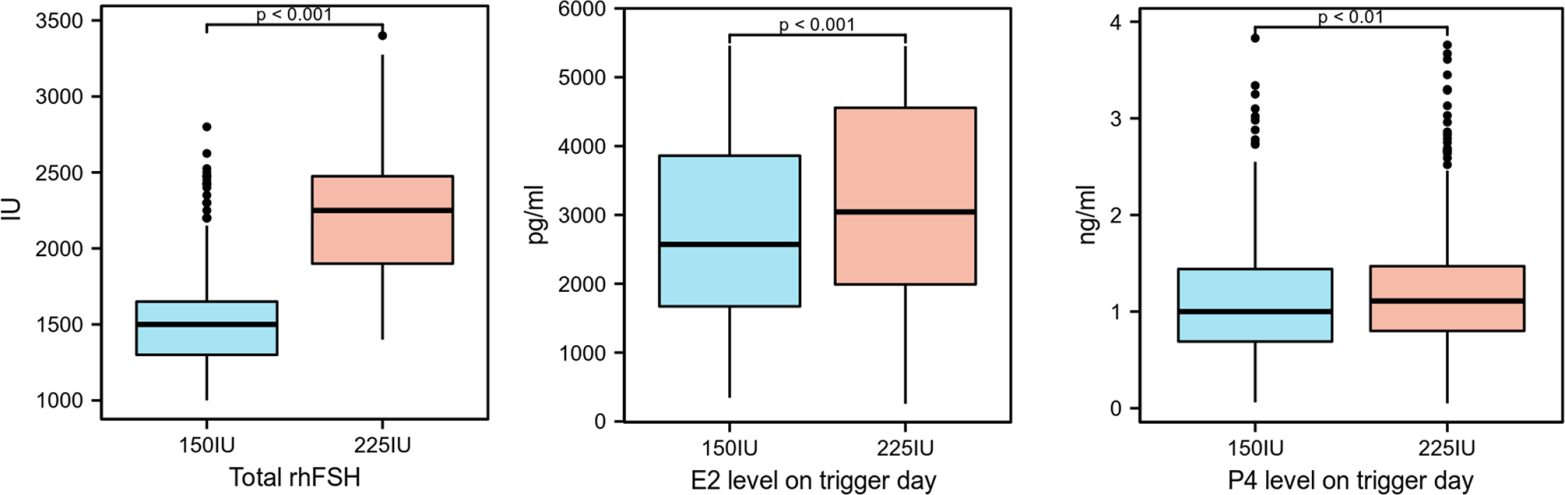
Statistically significant indicators of ovarian stimulation outcomes.

The characteristics of ovarian stimulation are presented in **Table 2**. The total dose of rhFSH in the 150 IU group was significantly lower than that in the 225 IU group (1517.63 ± 283.65 vs. 2218.62 ± 402.61, p<0.01) (**Figure 2**), and there was no difference in the stimulation duration of rhFSH administration. There was no difference in the total dose and duration of Cetrorelix between the two groups. The lag time from ovulation trigger to oocyte aspiration and ovulation trigger protocol, including hCG or GnRH agonist combined hCG, was not significantly different between the two groups. On trigger day, the 225 IU group had significantly higher levels of estradiol (2790.51 ± 1329.37 vs. 3139.98 ± 1403.4, p<0.01) and progesterone (1.11 ± 0.57 vs. 1.2 ± 0.6, p<0.01) than the 150 IU group (**Figure 2**). There was no severe OHSS in both groups, and mild to moderate OHSS was not statistically significant (3.70% vs 5.40%, p=0.181).

**Table 2:** Ovarian stimulation outcomes between the two groups.

**Groups**	**150 IU (n = 617)**		**225 IU (n = 521)**		**P-value**
Stimulation duration of rhFSH (day)	9.71 ± 1.63		9.85 ± 1.75		0.19
Total rhFSH (IU)	1517.63 ± 283.65		2218.62 ± 402.61		**p<0.01**
Duration of Cetrorelix (day)	5.81 ± 1.52		5.93 ± 1.62		0.18
Total Cetrorelix (mg)	1.45 ± 0.38		1.48 ± 0.41		0.18
LH level on trigger day (IU/L)	2.72 ± 1.96		2.88 ± 2.25		0.2
E2 level on trigger day (pg/ml)	2790.51 ± 1329.37		3139.98 ± 1403.4		**p<0.01**
P4 level on trigger day (ng/ml)	1.11 ± 0.57		1.2 ± 0.6		**p<0.01**
Ovulation trigger protocol					0.744
dual trigger	140/617 (22.70%)		114/521 (21.90%)		
Triggered with hCG	477/617 (77.30%)		407/521 (78.10%)		
Lag time from ovulation trigger to oocyte aspiration	36.11 ± 0.53		36.11 ± 0.55		0.899
OHSS	23/617 (3.70%)		28/521 (5.40%)		0.181

rhFSH, recombinant human follicle-stimulating hormone; LH, luteinizing hormone; E2, estradiol; P4, progesterone; OHSS, ovarian hyperstimulation syndrome. Data are presented as mean ± SD, median (IQR) and n (%). The bold font indicates that the p value is less than 0.05, which means it has statistical significance.

### 3.3 Embryological outcomes

**Figure 3:**
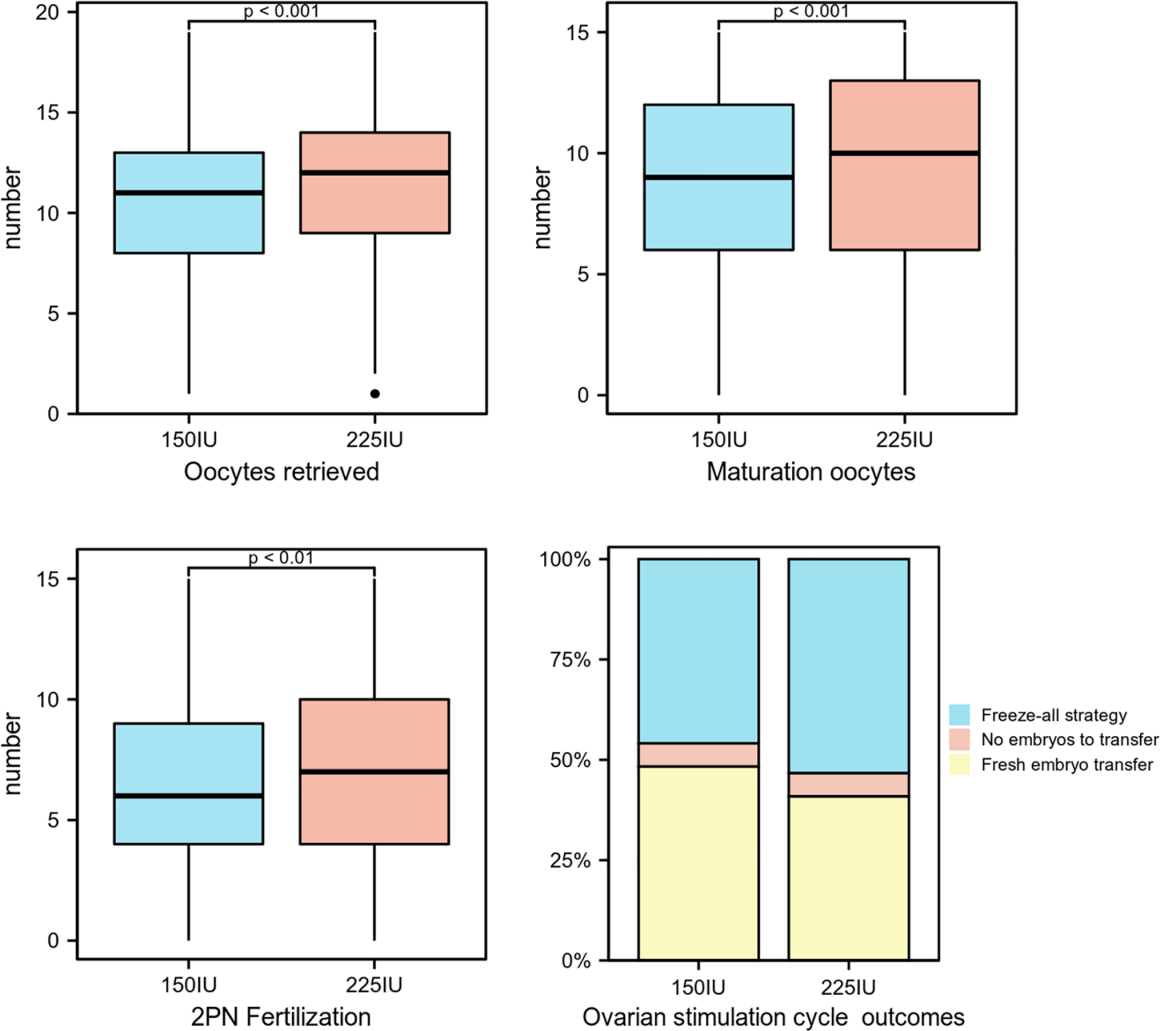
Statistically significant indicators of embryological outcomes.

The characteristics of the embryological are presented in **Table 3**. The number of oocytes retrieved (10.19 ± 3.6 vs. 11.16 ± 3.67, p<0.01), maturation oocytes (8.63 ± 3.91 vs. 9.6 ± 3.97, p<0.01), and two-pronuclear (2PN) fertilization (6.45 ± 3.52 vs. 7.16 ± 3.7, p<0.01) in the 150 IU group were significantly lower than those in the 225 IU group (**Figure 3**). There were no significant differences in the number of available embryos, number of high-quality embryos, proportion of high-quality embryos, number of blastocysts, and proportion of blastocysts between the two groups. For ovarian stimulation cycle outcomes, the proportion of fresh embryo transfer in the 150 IU group was significantly higher than that in the 225 IU group (48.30% vs. 40.90%, p=0.035) (**Figure 3**).

**Table 3:** Embryological outcomes between the two groups.

Groups	150 IU (n = 617)	225 IU (n = 521)	P-value
Number of oocytes retrieved	10.19 ± 3.6	11.16 ± 3.67	**p<0.01**
Maturation oocytes	8.63 ± 3.91	9.6 ± 3.97	**p<0.01**
2PN Fertilization	6.45 ± 3.52	7.16 ± 3.7	**p<0.01**
Number of available embryos	3.95 ± 2.61	4.23 ± 2.72	0.08
Number of high-quality embryos	1.34 ± 1.81	1.35 ± 1.68	0.96
Proportion of high-quality embryos	829/2440 (34.00%)	703/2204 (35.60%)	0.13
Number of blastocysts	1.32 ± 2.41	1.51 ± 2.83	0.23
Proportion of blastocysts	813/2440 (33.30%)	785/2204 (31.90%)	0.10
Ovarian stimulation cycle outcomes			**0.035**
Freeze-all strategy	283/617 (45.90%)	278/521 (53.40%)	
No embryos to transfer	36/617 (5.80%)	30/521 (5.80%)	
Fresh embryo transfer	298/617 (48.30%)	213/521(40.90%)	
Endometrial thickness prior to embryo transfer.	10.84 ± 2.02	10.68 ± 2.02	0.19
Number of embryos transferred			0.218
Single	54/298 (18.10%)	48/213 (22.50%)	
Double	244/298 (81.90%)	165/213 (77.50%)	
Quality of transferred embryos			0.421
Available embryos	131/298 (44.00%)	96/213 (45.10%)	
A high-quality embryo	141/298 (47.30%)	105/213 (49.30%)	
Two high quality embryos	26/298 (8.70%)	12/213 (5.60%)	
Blastocyst transfer	22/298 (7.40%)	24/213 (11.30%)	0.13

2PN, two-pronuclear. Data are presented as mean ± SD, median (IQR) and n (%). The bold font indicates that the p value is less than 0.05, which means it has statistical significance.

### 3.4 Pregnancy and birth outcomes

As demonstrated in **Table 4**, between-group comparisons in both 150 IU and 225 IU groups revealed insignificant differences in positive pregnancy per embryo transfer, biochemical pregnancy loss per positive pregnancy, clinical pregnancy per embryo transfer, ectopic pregnancy per positive pregnancy, ongoing pregnancy per embryo transfer, miscarriage per clinical pregnancy, live birth per embryo transfer, number of live births (all P > 0.05).

**Table 4:** Pregnancy and birth outcomes between the two groups.

Groups	150 IU (n = 298)	225 IU (n = 213)	P-value
Positive pregnancy per embryo transfer	153/298 (51.30%)	106/213 (49.80%)	0.725
Biochemical pregnancy loss per positive pregnancy	10/153 (6.50%)	9/106 (8.50%)	0.553
Clinical pregnancy per embryo transfer	143/298 (48.00%)	97/213 (45.50%)	0.585
Ectopic pregnancy per positive pregnancy	11/153 (7.20%)	6/106 (5.70%)	0.625
Ongoing pregnancy per embryo transfer	132/298 (44.30%)	91/213 (42.70%)	0.724
Miscarriage per clinical pregnancy	21/143 (14.70%)	13/97 (13.40%)	0.780
Live birth per embryo transfer	122/298 (40.90%)	84/213 (39.40%)	0.733
Number of live births			0.794
singletons	91/122 (74.60%)	64/84 (76.20%)	
twins	31/122 (25.40%)	20/84 (23.80%)	

A binary logistic regression model was also used to assess the association between starting dose of rhFSH and pregnancy and birth outcomes while adjusting for potential confounders (**Table 5**). Furthermore, in the crude and adjusted models, the 150 IU group was comparable to the 225 IU group in terms of positive pregnancy, clinical pregnancy, ongoing pregnancy, and live birth (all P > 0.05).

**Table 5:** Binary logistics regression analysis with pregnancy and birth outcomes as the influencing factor.

	starting dose of rhFSH	Crude model		Adjusted model	
		OR (95% CI)	P-value	OR (95% CI)	P-value
Positive pregnancy	150 IU	Reference		Reference	
	225 IU	1.065 (0.749-1.514)	0.725	1.02 (0.711-1.464)	0.915
Clinical pregnancy	150 IU	Reference		Reference	
	225 IU	1.103 (0.775-1.57)	0.585	1.074 (0.748-1.542)	0.699
Ongoing pregnancy	150 IU	Reference		Reference	
	225 IU	1.066 (0.748-1.52)	0.724	1.036 (0.719-1.491)	0.85
Live birth	150 IU	Reference		Reference	
	225 IU	1.065 (0.743-1.524)	0.733	1.043 (0.721-1.51)	0.822

CI, confidence interval.

Analyses were adjusted for age, BMI, AMH, infertility type (primary or secondary), starting dose of rhFSH, ovulation trigger protocol, total dose of Cetrorelix.

## 4 Discussion

So far, few studies have evaluated different starting dose of rhFSH on pregnancy outcomes for NOR patients in GnRH antagonist protocol. In this study, we qualified the NOR population in terms of age, FSH, AMH, and AFC following the expert consensus issued by CMDA and compared the IVF and pregnancy outcomes of the recommended starting dose of rhFSH in GnRH antagonist protocol in the consensus.

Compared to previous research, our practice can provide evidence-based guidance to select a starting dose of either 150 IU or 225 IU rhFSH for IVF based on *post-hoc* randomization and a large sample for the NOR population in GnRH antagonist protocol. Data from this study suggest that in NOR patients, a starting dose of rhFSH of 225 IU has no advantage over 150 IU in terms of pregnancy and live birth outcomes.

The dose-dependent increase between the number of retrieved oocytes and rhFSH has been confirmed. For the number of oocytes retrieved, the 225 IU group was significantly more than the 150 IU group (11.16 ± 3.67 vs. 10.19 ± 3.6, p<0.01), which is consistent with the results of a published prospective randomized controlled trial. In a randomized, double-blind, multicenter clinical trial comparing starting dose of 150 and 200 IU of rhFSH, the results showed that the 200 IU group had an average of 0.6 more oocytes than the 150 IU group (16). In another prospective randomized study comparing daily doses of 150 and 225 IU, the results showed that the 225 IU group had an average of 1.9 more oocytes than the 150 IU group (17). However, the patient in these studies included advanced maternal age, and there is a consensus that women over 35 are defined as advanced maternal age. Also, the sample sizes of the two articles were small, and pregnancy and live birth outcomes were not followed up.

In studies using higher rhFSH over 225 IU, the results indicated no significant differences in the number of oocytes retrieved and pregnancy outcomes (18). The mild increase in the number of oocytes retrieved in the high-dose group did not even increase the number of available embryos, either in our study or in previous studies.

In addition, no improvement in pregnancy outcomes was found in any of these studies. Therefore, the value of increasing the total number of retrieved oocytes should not be overemphasized for NOR patients.

GnRH analogues inhibit endogenous LH surges and prevent early follicular ovulation and follicular luteinization. Despite the use of GnRH analogs during COS, Premature progesterone elevation still occurred due to high doses of rhFSH, high E2 levels, and the simultaneous development of multiple follicles (19, 20). Meanwhile, the simultaneous development of multiple follicles leads to an increase in serum estradiol levels during COS (21). In our study, progesterone levels were significantly higher in the 225 IU group than in the 150 IU group on the trigger day (1.11 ± 0.57 vs. 1.2 ± 0.6, p=0.01).

Two large retrospective cohort studies showed a negative impact on pregnancy outcomes in women undergoing IVF when progesterone > 1.5-2 ng/mL (22, 23).

The putative negative effect of premature progesterone elevation is embryo-endometrial asynchrony which is critical for successful implantation (24).

Elevated progesterone levels on the trigger day were negatively correlated with live births in the fresh embryos transfer cycle but not in the subsequent frozen embryo transfer cycles (25, 26). Therefore, the freeze-all strategy was performed to reduce the effect of elevated progesterone on live birth rates when progesterone exceeds 1.5-2ng/ml (27). In this study, the freeze-all strategy was significantly higher in the 225 IU group than in the 150 IU group, which was associated with more patients in the 225 IU group having elevated progesterone. A systematic review showed that the freeze-all strategy was not superior to fresh embryo transfer in terms of live birth rate (28, 29). Conversely, the risk of maternal hypertensive disorders of pregnancy, of having a large-for-gestational-age baby and a higher birth weight of the children born may be increased following the freeze-all strategy (30). By design, the time to pregnancy is shorter in the conventional strategy than in the freeze-all strategy when the cumulative live birth rate is comparable, as embryo transfer is delayed in a ‘freeze-all’ strategy (31). At the same time, the freeze-all strategy increases the financial burden on patients (32). Therefore, the freeze-all strategy is unsuitable for all patients (33). The fresh embryo transfer should be adopted as much as possible without affecting live births, considering clinical safety and convenience (34).

OHSS is a potentially life-threatening iatrogenic complication during COS (35). A study analyzing 256,381 *in vitro* fertilization cycles showed that retrieval of >15 oocytes significantly increase OHSS risk during COS (36). In our study, we excluded patients with a high prevalence of OHSS, such as those with polycystic ovary syndrome, and limited the number of AFC in the patients. Therefore, there was no severe OHSS occurring between the two groups, and mild to moderate OHSS was not statistically significant.

Live birth is the principal clinical outcome following IVF. Previous studies have shown that when the total dose of rhFSH is more than 2500 IU, it negatively affects the live birth rate in fresh embryo transfer (37). This suggests that the CMDA consensus recommendation of a starting rhFSH dose of 150 or 225 IU is equally safe and reliable for NOR patients. In our study, the total dose of rhFSH dose did not exceed this upper limit in either group. Also, there was no difference in the live birth rate between the two groups in fresh embryo transfer. This suggests that a starting dose of rhFSH of 150 or 225 IU is equally safe and reliable for NOR patients.

A potential limitation of the study is that adjustments in rhFSH dose were permitted during COS (38), with adjustments limited to 50 IU. The dose adjustment of rhFSH is part of daily clinical practice during COS, and a systematic review covering 10 years showed that the proportion was about 45% (39). In addition, the dose adjustment of rhFSH did not affect the live birth (40). In spite of all the efforts to control bias, this study is inherently limited by the review of a retrospectively collected data set. Despite these limitations, our study still provides clinicians with a reasonable option for starting dose of rhFSH in the NOR population.

## 5 Conclusion

In conclusion, for the NOR patients following the GnRH-ant protocol, the starting dose of rhFSH of 225 IU slightly increased the number of oocytes retrieved compared to the starting dose of rhFSH of 150 IU, at the cost of an extra approximate 700 IU of rhFSH during COS. However, there was no significant difference in the number of available embryos and live birth rate in fresh embryo transfer. Notably, the fresh embryo transfer rate was higher in the 150 IU group than in the 225 IU group. Considering the potential cost-effectiveness and shorter time to live birth, the starting dose of rhFSH of 150 IU may be more suitable than 225 IU.

## Data availability statement

The raw data supporting the conclusions of this article will be made available by the authors, without undue reservation.

## Ethics statement

The study was authorized by the local institutional review board (Reproductive Ethics Committee of The Affiliated Hospital of Shandong University of Traditional Chinese Medicine, approval no. SDTCM/E2204-02, dated April 2, 2022) and was undertaken at a public tertiary referral university hospital.

## Author contributions

Z-CJ collected data and developed the manuscript. Y-QL and RL guided the design and reviewed the manuscript. SH, Q-CX, and KY contributed to data collection. P-XW and S-ML assisted in data analysis. Z-GS and YG guided the design and implementation of the study. All authors contributed to the article and approved the submitted version.
